# Acute effects of combined exercise and oscillatory positive expiratory pressure therapy on sputum properties and lung diffusing capacity in cystic fibrosis: a randomized, controlled, crossover trial

**DOI:** 10.1186/s12890-018-0661-1

**Published:** 2018-06-14

**Authors:** Thomas Radtke, Lukas Böni, Peter Bohnacker, Marion Maggi-Beba, Peter Fischer, Susi Kriemler, Christian Benden, Holger Dressel

**Affiliations:** 1 Epidemiology, Biostatistics and Prevention Institute (EBPI), University of Zurich, Zurich, Switzerland; 2 Division of Occupational and Environmental Medicine, University of Zurich and University Hospital Zurich, Zurich, Switzerland; 3 Department of Health Science and Technology, ETH Zurich, Zurich, Switzerland; 4 Division of Pulmonology, University Hospital of Zurich, Zurich, Switzerland

**Keywords:** Lung disease, Sputum viscoelasticity, Diffusing capacity for nitric oxide, Exercise, Airway clearance, Mucus

## Abstract

**Background:**

Regular airway clearance by chest physiotherapy and/or exercise is critical to lung health in cystic fibrosis (CF). Combination of cycling exercise and chest physiotherapy using the Flutter® device on sputum properties has not yet been investigated.

**Methods:**

This prospective, randomized crossover study compared a single bout of continuous cycling exercise at moderate intensity (experiment A, control condition) vs a combination of interval cycling exercise plus Flutter® (experiment B). Sputum properties (viscoelasticity, yield stress, solids content, spinnability, and ease of sputum expectoration), pulmonary diffusing capacity for nitric oxide (*D*LNO) and carbon monoxide (*D*LCO) were assessed at rest, directly and 45 min post-exercise (recovery) at 2 consecutive visits. Primary outcome was change in sputum viscoelasticity (G’, storage modulus; G”, loss modulus) over a broad frequency range (0.1–100 rad.s^− 1^).

**Results:**

15 adults with CF (FEV_1_range 24–94% predicted) completed all experiments. No consistent differences between experiments were observed for G’ and G” and other sputum properties, except for ease of sputum expectoration during recovery favoring experiment A. *D*LNO, *D*LCO, alveolar volume (V_A_) and pulmonary capillary blood volume (V_cap_) increased during experiment A, while *D*LCO and V_cap_ increased during experiment B (all *P* < 0.05). We found no differences in absolute changes in pulmonary diffusing capacity and its components between experiments, except a higher V_A_ immediately post-exercise favoring experiment A (*P* = 0.032).

**Conclusions:**

The additional use of the Flutter® to moderate intensity interval cycling exercise has no measurable effect on the viscoelastic properties of sputum compared to moderate intensity continuous cycling alone. Elevations in diffusing capacity represent an acute exercise-induced effect not sustained post-exercise.

**Trial registration:**

ClinicalTrials.gov; No.: NCT02750722; URL: clinical.trials.gov; Registration date: April 25th, 2016.

**Electronic supplementary material:**

The online version of this article (10.1186/s12890-018-0661-1) contains supplementary material, which is available to authorized users.

## Background

Cystic fibrosis (CF) is the most common genetic life-limiting disease in Caucasians caused by abnormalities in CF conductance transmembrane protein function. Depletion of airway surface liquid, dehydrated mucus, chronic inflammation and infection contribute to accumulation of secretions and subsequent progressive lung damage in CF [1]. Regular airway clearance is therefore of critical importance to lung health in CF. Exercise and chest physiotherapy are accepted airway clearance techniques (ACT’s) in CF, belonging to the current top ten research priorities [2]. There are a number of different ACT’s such as the active cycle of breathing technique, high-frequency chest wall oscillations, positive expiratory pressure (PEP) and oscillatory PEP. Oscillatory PEP with the Flutter® is equally effective compared to other ACT’s [3] and has been shown to favorably alter respiratory flow [4], to increase sputum expectoration [5] and to reduce sputum viscoelasticity [6, 7] in CF.

Exercise alone or in combination with chest physiotherapy improves airway clearance and sputum expectoration [6, 8], but the exact mechanisms are not fully understood. Moreover, acute exercise is thought to facilitate mucociliary clearance by increased shear stress on airway epithelium and ciliary beat frequency as a result of an increased ventilation [9], improved water content of mucus [10] and increased trunk oscillations during weight bearing exercises such as walking or running [11]. Recently, moderate intensity exercise has been identified as ‘optimal training intensity’ for individuals with mild to moderate CF lung disease due to improvements in bronchodilation and pulmonary diffusing capacity and prevention of airflow restriction compared to vigorously intense exercise [12]. Enhanced mucus clearance might potentially (at least in part) result in an improvement in pulmonary gas diffusion.

The aim of this study was to compare a single bout of moderate intensity cycling exercise incorporating a breathing therapy device, the Flutter®, with a single bout of moderate intensity cycling exercise alone on sputum viscoelasticity (primary endpoint) and pulmonary diffusing capacity in adults with CF. We hypothesized that the combination of a high ventilation during cycling exercise in combination with increased oscillatory shear stress (Flutter®) yields greater improvements in sputum viscoelasticity compared to cycling exercise alone.

## Methods

### Study design

**Fig. 1:**
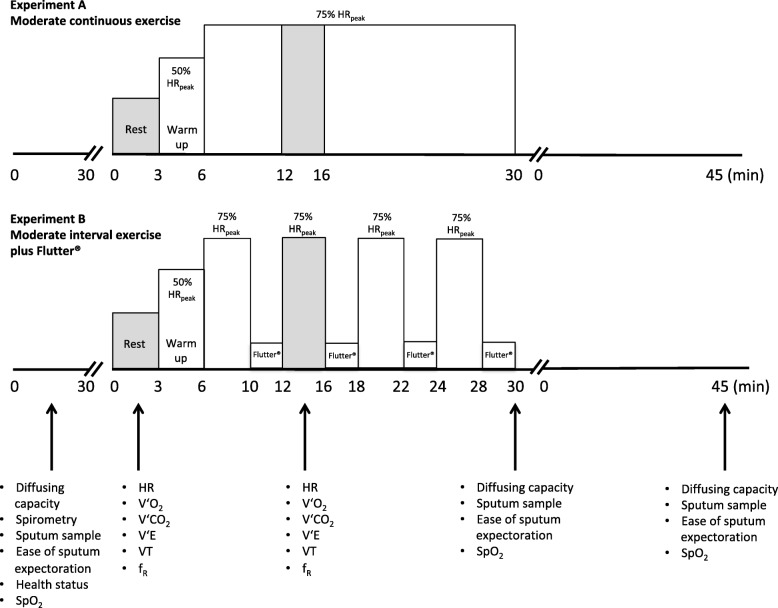
Experimental study design. f_R_, respiratory frequency; HR, heart rate; SpO_2_, oxygen saturation; V’CO_2_, carbon dioxide production; V’E, minute ventilation; V’O_2_, oxygen consumption; VT, tidal volume. The grey shaded area represents the periods when the participants breathed through the mouthpiece for metabolic cart measurements

We conducted a prospective, randomized, controlled crossover trial (Clinicaltrials.gov, NCT02750722). Adults with CF were invited to our laboratory facility on three different occasions. At the first study visit, the patients provided a sputum sample, performed pulmonary function testing and cardiopulmonary exercise testing (CPET). At the second and third study visit, patients provided sputum samples, performed pulmonary function testing and, depending on randomization, performed either continuous cycling exercise at moderate intensity without Flutter® (Experiment A, control condition) or moderate intensity interval cycling exercise incorporating Flutter® therapy (Experiment B, experimental condition). The detailed exercise protocol including measurements and assessments during study visit 2 and 3 are shown in Fig. 1.

### Study participants

Individuals with CF were recruited from the Adult CF Center at the University Hospital Zurich, Switzerland, between June 2016 and January 2017. Patients aged 18 years and older with a confirmed diagnosis of CF able to provide sputum samples were included. Exclusion criteria were as follows: i) listing for lung transplantation or status post lung transplantation, ii) chronic pulmonary infection with *Burkholderia* cepacia complex, iii) unstable clinical condition (i.e., major hemoptysis or pneumothorax within the last 3 months, acute pulmonary exacerbation [13], intravenous antibiotic treatment during the last 4 weeks, change in pulmonary medication during the study period); iv) cardiac arrhythmias with exercise and v) requirement of additional oxygen with exercise. Ethical approval was obtained from the Cantonal Ethics Committee of Zurich (2015–00153), Switzerland. All patients provided written informed consent.

### Randomization

We used central randomization to randomly allocate patients to the different experimental conditions (Experiment A or B). A person not involved in the study generated a list of random numbers using the statistical software package STATA (Version 12, StataCorp. 2012, College Station, Texas, USA). The list consisted of 30 random numbers, where even numbers represented the experimental condition ‘A’ and odd numbers the condition ‘B’. The order of the experiments at study visit 2 and 3 was either ‘A-B’ or ‘B-A’. At the first study visit, after written informed consent was obtained, the principal investigator (TR) called the person creating the list of random numbers to obtain information on the order of the exercise experiments for visit 2 and 3, respectively. The patient was immediately informed about the testing order for the following study visits.

### Assessments

All tests were scheduled at the same time of the day (+ − 1 h deviation) to avoid any potential impact of diurnal variation on pulmonary function measurements [14]. The time period between study visit 1 and 3 was 8 ± 2 days, respectively. Patients were told to abstain from fatty meals (for 3 h), caffeine-containing substances (for 4 h) and to avoid vigorous physical exercise during the last 24-h prior to the study visits, respectively. Moreover, patients were told to abstain from their regular inhalation and airway clearance therapy on the day of the study visits. The following provides a short description of assessments methods.

#### Sputum samples

At the beginning of each study visit, one sputum sample was collected. At study visits 2 and 3, two additional samples were collected immediately post-exercise and again 45 min post-exercise, referred to as recovery (Fig. 1). Sputum was gently expectorated and collected into sterile and coded containers (cryotubes 5 mL, VWR). The containers were immediately stored at − 4 °C and transferred on ice into a deep freezer (− 80 °C) after each study visit.

#### Sputum rheology

Rheological measurements were performed on a MCR 702 rheometer (Anton Paar, Austria) in parallel plate mode, using sandblasted 25 mm diameter stainless steel plates (PP25 S Anton Paar, Austria) and a gap size of 0.5 mm. First, a frequency sweep was performed (0.1–100 rad s^− 1^, γ = 1%) followed by an amplitude sweep (0.1–1000%, ω = 1 rad s^− 1^). The snap frozen sputum samples (− 80 °C) were transferred to the fridge (4 °C) at least 6 h before the measurement. The slowly thawed samples were then transferred from the cryotubes to the lower measuring plate using a 1 mL micropipette. The micropipette tips were cut in the front with a scalpel to have a larger die, thus minimizing shear on the sample. The upper plate was slowly lowered onto the sputum, and a solvent trap containing moist sponges was placed over the sample. Prior to measurements, the sputum was let to rest for five minutes. All measurements were performed temperature controlled at 20 °C. Inertia calculations are based on equations reported by Ewoldt et al. [15].

**Fig. 2:**
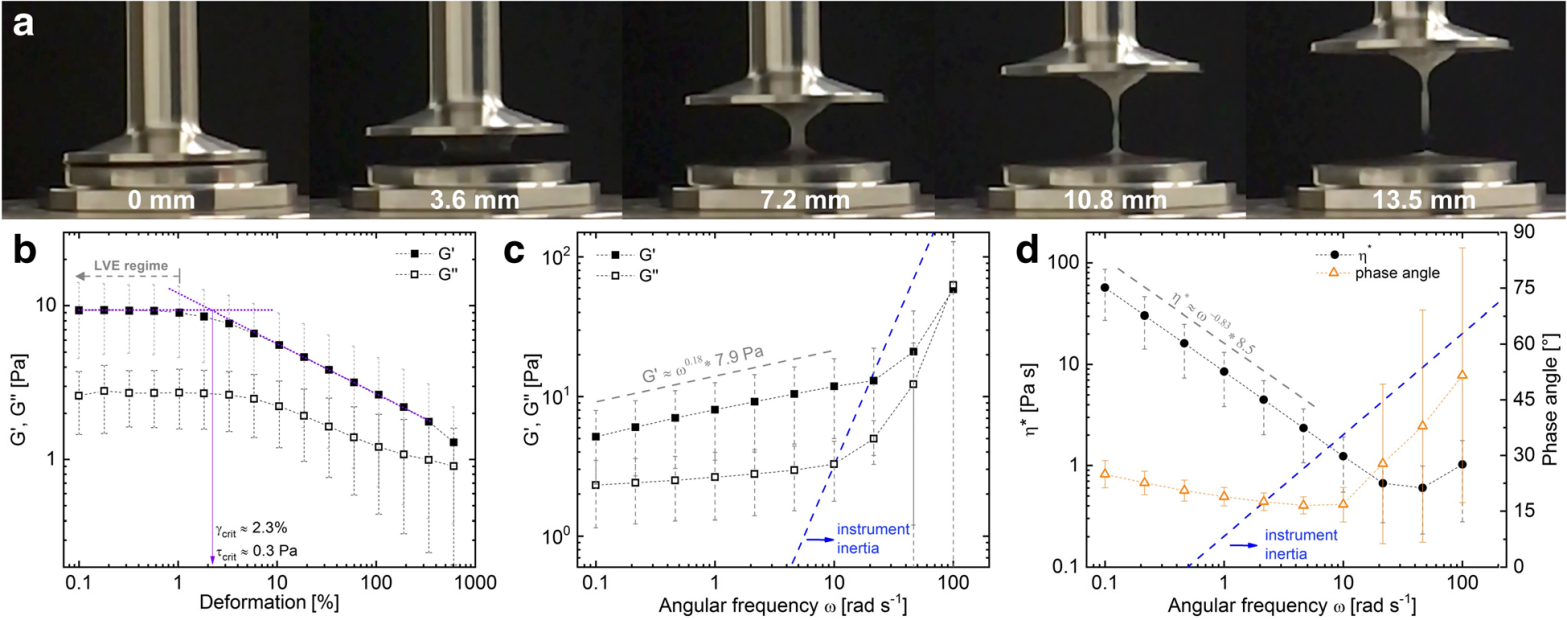
Rheology and spinnability of cystic fibrosis sputum. (**a**) Spinnability of sputum was assessed at the end of rheological measurements by lifting up the upper plate at a constant velocity of 3.6 mm s^− 1^. (**b**) Amplitude sweep (at 1 rad s^− 1^) showing a linear viscoelastic (LVE) regime up to about 1% deformation. The dashed violet lines show the graphical determination of the dynamic yield point / stress. (**c**, **d**) Frequency sweeps depicting G’ (storage modulus), G” (loss modulus) and η^*^ (complex viscosity). The grey dashed lines represent the vertically shifted fitting curves (fitting between 0.1–10 rad s^− 1^). The blue dashed lines in (**b**, **c**) indicate the calculated instrument inertia limit

The spinnability of sputum describes its ability to form filaments, which provides valuable information about cohesion forces in the sputum [16]. At the end of each rheological measurement, the upper plate was separated from the lower plate at a constant velocity of 3.6 mm s^− 1^ [17], which was filmed (iPhone 6, Apple) at 120 fps (Fig. 2a). The sputum formed a filament between the separating plates. The separation distance at which the sample broke (‘spinnability’) was extracted from the movie by counting the amount of frames from the onset of separation until filament breaking.

To estimate sputum solids content, 0.25 mL aliquots of sputum were filled in 1.5 mL HPLC vials (VWR, Switzerland) and weighed with a high precision scale (Mettler AE 163, Mettler Toledo, Switzerland). The samples were then dried for 24 h at 50 °C and a pressure of 100 mbar using a vacuum drying oven (SalvisLab, Switzerland) and subsequently weighed again. All rheological measurements were done by a person (PB) who was blinded with respect to the two different experimental conditions.

Ease of sputum expectoration was assessed on a 10 cm visual analogue scale (0 = very difficult and 10 = very easy). A blinded assessor, not involved in the study, measured the distance for all scales after completion of all study experiments.

#### Spirometry

Spirometry was always performed before diffusing capacity measurements with the patient in sitting position using a commercially available system (MasterScreen™ PFT Pro, Jaeger, PanGas AG Healthcare, Switzerland) according to American Thoracic Society/European Respiratory Society Standards [18]. All tests were performed pre-bronchodilation (i.e., withheld of short-acting bronchodilators and anticholinergic drugs for at least 4 h, long-acting bronchodilators for at least 12 h, and once-daily, long-acting bronchodilators for at least 24 h). We calculated percent-predicted values for forced expiratory volume in 1 s (FEV_1_) using reference equations from Quanjer et al. [19].

#### Pulmonary diffusing capacity

Details on the methods can be found elsewhere [20]. In brief, pulmonary diffusing capacity measurements were done in triplicate at rest and for the measurements done 45 min post-exercise (visit 2 and 3). If intra-session reproducibility criteria were not fulfilled additional measurements were performed [21]. Two maneuvers were performed immediately post-exercise (see Fig. 1 in the publication). In general, a 5 min pause was done between consecutive maneuvers to assure adequate elimination of test gas from the lungs [22]. For the analysis, we used the average value of the first two single-breaths tests for maneuvers performed at rest and 45 min post-exercise, when intra-session acceptability criteria were fulfilled [21]. If intra-session acceptability criteria were not fulfilled [21], the third single-breath test was considered and the average of the two highest test results was used. For the measurements immediately post-exercise (see Fig. 1 in the publication), the first test was used for statistical analysis. We calculated percent-predicted values for diffusing capacity for nitric oxide (*D*LNO) and carbon monoxide (*D*LCO), pulmonary capillary blood volume (V_cap_) and the alveolar-capillary membrane diffusing capacity for carbon monoxide (*D*MCO) according to references equations published by Zavorsky et al. [21].

#### Cardiopulmonary exercise testing

Cardiopulmonary exercise testing (CPET) was performed on a cycle ergometer (custo ec 3000e, custo med GmbH, Ottobrunn, Germany) using the Godfrey Protocol [23]. The test started with a three-minute rest period followed by three-minutes of unloaded pedaling at 60–70 rpm. Afterwards, the workrate (W, watts) was increased every minute according to the patient’s height: 10 W (< 120 cm), 15 W (120–150 cm) or 20 W (> 150 cm) [23]. The increment was individually adapted for patients with severely reduced lung function (i.e., FEV_1_ < 30% predicted). After the patients had reached their maximal exercise performance, he/she rested for another three-minutes on the cycle ergometer (recovery phase).

The metabolic cart (Metalyzer®, Cortex Biophysik GmbH, Leipzig, Germany) was calibrated with gases of known standard concentrations before each test. Heart rate was measured with a Polar heart rate monitor (Polar RS400, Polar Electro, Oy, Kempele, Finland) and oxygen saturation (SpO_2_) was continuously measured at the earlobe (Nonin® Xpod® PureSAT®,Nonin Medical, Inc, USA). Ratings of perceived exertion and dyspnea were evaluated at peak exercise by means of a 0–10 Borg scale [24]. One of the following criteria had to be fulfilled to ensure the test was maximal: 1) plateau in oxygen consumption (V’O_2_) despite an increase in workrate; 2) peak heart rate over 85% of predicted [25], 3) respiratory exchange ratio (RER) > 1.05, 4) peak ventilation exceeded predicted maximum voluntary ventilation (calculated as FEV_1_ × 35) and 5) subjective impression of the supervisor. Maximal heart rates were used to calculate exercise intensities (50 and 75% of peak heart rate) for the cycling experiments at visit 2 and 3.

#### Patient-reported health status

Patient-reported health status was assessed with the Feeling Thermometer. The Feeling Thermometer is part of the EQ-5D, a common instrument used for healthy economic analyses and established by the EuroQol group [26]. The Feeling Thermometer is a modified visual analogue scale in form of a thermometer. The instrument has marked intervals from 0 (worst health state ) to 100 (perfect health). We used the instrument to evaluate whether patients were in a stable health condition during the study.

#### Anthropometry

We measured each patient’s height to the nearest 0.1 cm using a stadiometer (Seca). Body weight was measured to the nearest 0.5 kg at each study visit using a balanced scale (Seca, Model 791, Vogel & Halke).

#### Moderate cycling exercise with and without Flutter® therapy

At study visit 2 and 3, moderate intensity cycling exercise at 75% of the peak heart rate achieved during CPET was performed either continuously (experiment A) or in 4-min intervals (experiment B) interspersed with 2-min resting periods, during which breathing maneuvers were performed using the Flutter® (see Fig. 1). At rest and during cycling (minute 12–16′), patients respired through a mouthpiece connected to the metabolic cart to measure respiratory gases (Fig. 1). Heart rate was continuously monitored with a chest belt and heart rate monitor (Polar RS400, Polar Electro, Oy, Kempele, Finland) and SpO_2_ with an earlobe pulse oximeter (Nonin® Xpod® PureSAT®,Nonin Medical, Inc, USA), respectively. The average value during 3 min at rest and during minutes 14–16 during exercise (steady-state conditions) were used for data analyses.

#### Flutter® breathing therapy

The Flutter® (VRP1, Eur. Patent. No: 0337990) is an airway clearance device providing oscillations during exhalation and vibrations of the airways aiming to facilitate mucus clearance in the airways. The Flutter® is a pipe like device with an oscillating stainless steel ball and a perforated cover. The device produces positive expiratory pressure and the angle at which the Flutter® is held determines the oscillation frequency of 6 to 20 Hz [27]. Exhalation through the Flutter® causes oscillation of the steel ball and produces rhythmic variations in positive expiratory pressure of 10 to 25 cm H_2_O [28, 29]. Flutter® therapy increases peak expiratory flow and creates an expiratory airflow bias in CF [6], initiating mucociliary clearance mechanisms.

During experiment B, 6–10 breathing maneuvers were performed during each of the 2 min resting periods (Fig. 1) without forced expiratory technique. The breathing maneuvers started with a slightly deep inspiration, a 2–3 s end-inspiratory pause and a forced expiration lasting about 5 s while the patient was sitting on the cycle ergometer without pedaling. The Flutter® device was kept in neutral (horizontal) position to maximize the oscillation amplitude and to target a frequency range of 10–15 Hz [29], optimal for mucus clearance. All patients received proper instructions on the use of the device at the first study visit. Patients without experience with the Flutter® received a device at the end of visit 1 enabling practice at home before the second study visit.

#### Statistical analysis and sample size calculation

All statistical analyses were performed with the statistical software package SPSS version 23 (IBM Corp. Armont, NY, USA). Descriptive data are presented as median (interquartile range, IQR), mean ± SD or N (%). We used the non-parametric Friedman Test to test for differences in resting (pre-exercise) sputum properties, spirometry, pulmonary diffusing capacity and patient-reported health status between the three study visits. Differences in outcome variables between the three different time points (pre-exercise; immediately post-exercise and 45 min post-exercise, respectively) during each experimental condition were analyzed with the non-parametric Friedman test followed by a Wilcoxon-signed rank test, if changes over time in the Friedman test were significant (*P* < 0.05). We calculated absolute changes between the different time-points (i.e., post-exercise minus pre-exercise and 45 min post-exercise minus pre-exercise values) and compared the two experimental conditions using the non-parametric Mann-Whitney-U test. The level of statistical significance was set as *P* < 0.05.

No previous study has investigated changes in sputum viscoelasticity following a combination of cycling and Flutter® using the same instruments as in our study from which we could derive means and standard deviations (SD’s) and on which we could base our power calculations. However, two previous studies using randomized crossover designs comparing i) an acute bout of cycling and treadmill exercise versus no exercise [11] or ii) Flutter® therapy with autogenic drainage [7] were able to demonstrate significant changes in sputum viscoelasticity in 14 individuals with CF in each study. We therefore aimed to include 16 patients in our study.

## Results

Baseline patient characteristics are shown in Table 1 (*N* = 16).

**Table 1:** Patient baseline characteristics

Variables	
N	16
Sex (male/female)	7/9
Age (years)	23 (22, 25)
BMI (kg m^− 2^)	20 (18, 21)
Cystic fibrosis-related diabetes (*N* (%))	8 (50)
Pancreatic insufficiency (*N* (%))	13 (81)
Chronic *Pseudomonas aeruginosa* infection (*N* (%))	7 (44)
FEV_1_ (% predicted)	52 (43, 72)
*D*LNO (% predicted)	59 (51, 73)
*D*LCO (% predicted)	82 (69, 86)
V’O_2peak_ (mL kg^− 1^ min^− 1^)	32.0 (30.3, 34.4)
Mechanical power (W kg^− 1^)	2.9 (2.3, 3.1)
HR_peak_ (beats min^− 1^)	167 (163, 179)
RER	1.20 (1.15, 1.26)

Data are median (IQR) or N (%). *BMI* body mass index, *CF* cystic fibrosis, *DLCO* diffusing capacity of the lung for carbon monoxide, *DLNO* diffusing capacity of the lung for nitric oxide, *FEV*_*1*_ forced expiratory volume in one second, *HR*_*peak*_ peak heart rate, *RER* respiratory exchange ratio, *V’O*_*2peak*_ peak oxygen consumption

During the study, one female patient requiring oral antibiotic therapy for treatment of a pulmonary exacerbation and was excluded from the analyses. All other patients completed all assessments without complications. There were no differences in pre-exercise sputum properties, pulmonary function data and patient-reported health status between the three study visits, respectively (Additional file 1: Table S1). Cardiorespiratory variables during exercise were comparable between both experiments (Additional file 2: Table S2), whereas patients had a higher respiratory frequency during experiment A compared to B (*P* < 0.001).

All patients were able to provide sputum samples at the requested time points during the study. 13/15 patients had previous experience using Flutter®, but only two were using the Flutter® on a regular basis.

### CF sputum viscoelasticity and spinnability

The average of all sputum samples (*n* = 45) obtained at rest during each of the three visits showed spinnability (Fig. 2a) and viscoelastic behavior (Fig. 2b-d) characteristic for CF sputum [7, 30–32]. Details can be found in the online supplements (Additional file 3: Table S4). At high frequencies (ω > 10 rad s^− 1^) G’ and G” as well as η^*^ (complex viscosity) and the phase angle increased due to instrument inertia, which causes artifacts in the sample signal. Consequently, the analysis of sputum viscoelasticity (G’ and G”) was restricted to an angular frequency of 10 rad s^− 1^. Instrument inertia is further debated in the Discussion section and online supplements.

### Changes in sputum rheological properties

Table 2 shows changes in sputum properties for each experimental condition. No time-course changes were found during either experiment A or B, except for sputum solids content during experimental condition A (*P* = 0.038). Individual raw data for G’ at 1 and 10 rad s^− 1^ are shown in Additional file 4: Figure S1 and Additional file 5: Figure S2 in the online supplements. In the experimental condition A, one study participant had very high values for G’ at 1 and 10 rad s^− 1^ immediately post-exercise. Of note, there was no technical problem during the rheological measurements, but the sputum sample was purulent and thick. A summary of all sputum rheological data excluding this particular participant is shown in Additional file 6: Table S3 in the online supplements.

**Table 2:** Changes in sputum properties, pulmonary diffusing capacity and oxygen saturation during experiments A and B (*N* = 15)

	Experiment A				Experiment B			
Variables	Pre-exercise	Post-exercise	45 min post-exercise	*P*-value	Pre-exercise	Post-exercise	45 min post-exercise	*P*-value
Sputum properties								
G’ 1 rad s^−1^ (Pa)	6.7 (4.2, 9.7)	10.3 (6.0; 20.3)	14.1 (6.1, 19.0)	0.057	7.5 (4.1, 13.0)	8.8 (6.5, 13.8)	13.3 (7.1, 14.6)	0.085
G’ 10 rad s^−1^ (Pa)	10.2 (6.4, 14.4)	15.9 (8.3, 28.1)	20.9 (9.7, 26.0)	0.062	11.5 (5.9, 20.1)	13.6 (8.8, 18.0)	20.0 (10.1, 23.0)	0.155
G” 1 rad s^−1^ (Pa)	2.3 (1.7, 3.2)	3.1 (2.1, 5.9)	3.8 (2.0, 6.2)	0.085	2.6 (1.3, 3.6)	3.2 (2.3, 4.9)	4.5 (2.5, 4.9)	0.155
G” 10 rad s^−1^ (Pa)	2.9 (2.2, 4.0)	3.7 (2.6, 6.6)	4.2 (2.6, 6.7)	0.085	3.6 (1.8, 4.3)	3.7 (2.8, 5.7)	5.3 (2.9, 6.1)	0.282
Dynamic yield stress (Pa)	0.20 (0.10, 0.30)	0.30 (0.20, 0.60)	0.30 (0.20, 0.40)	0.066	0.20 (0.10, 0.40)	0.20 (0.18, 0.33)	0.30 (0.2, 0.53)	0.074
Sputum solids content (%)	5.3 (3.9, 7.4)	6.2 (4.2, 9.7)	6.5 (4.4, 8.1)	0.038	4.5 (3.6, 5.8)	6.2 (4.1, 7.5)	6.9 (3.8, 8.4)	0.672
Spinnability (mm)	6.7 (6.4, 8.2)	7.7 (6.5, 21.0)	6.7 (6.3, 8.2)	0.155	9.2 (6.4, 12.4)	6.2 (5.8, 8.3)	8.9 (7.2, 31.0)	0.089
Ease of sputum expectoration (cm)	8.1 (5.2, 8.3)	7.7 (3.4, 9.3)	8.2 (4.7, 8.9)	0.180	7.1 (5.2, 8.7)	6.5 (4.4, 9.1)	5.3 (4.3, 9.2)	0.482
Pulmonary diffusing capacity								
*D*LNO (mL min^− 1^ mmHg^− 1^)	78.1 (68.4, 141.6)	86.9 (67.4, 140.6)	79.2 (65.6, 134.0)	0.015^*^	84.7 (64.8, 143.1)	83.8 (69.9, 135.2)	82.6 (69.4, 147.8)	0.482
*D*LNO/V_A_ (mL min^− 1^ mmHg^− 1^ L^− 1^)	21.1 (19.8; 22.4)	21.2 (20.0; 22.2)	20.7 (18.2; 21.8)	0.015^♯^	21.1 (19.7; 22.7)	20.5 (19.2; 22.4)	20.7 (18.3, 22.3)	0.007^♯^
*D*LCO (mL min^− 1^ mmHg^− 1^)	22.4 (20.3, 31.9)	24.6 (20.3, 36.2)	21.5 (19.5, 31.1)	0.002^*^	23.7 (19.6, 34.3)	24.2 (19.4, 34.0)	22.9 (19.3, 32.5)	0.011^*^
*D*LCO/V_A_ (mL min^− 1^ mmHg^− 1^ L^− 1^)	5.8 (5.2, 6.1)	6.1 (5.4, 6.5)	5.6 (5.3, 6.2)	0.047^*^	5.7 (5.4, 6.01)	6.0 (5.3, 6.2)	5.5 (5.0, 5.9)	< 0.001^*♯^
*D*LNO/*D*LCO ratio	3.8 (3.4, 4.1)	3.6 (3.4, 3.8)	3.6 (3.4, 4.1)	0.025	3.8 (3.4, 4.2)	3.5 (3.3, 3.9)	3.7 (3.3, 4.1)	0.005
*D*MCO (mL min^−1^ mmHg^− 1^)	52 (42, 112)	56 (43, 88)	53 (43, 92)	0.612	58 (42, 106)	54 (45, 89)	56 (42, 118)	0.717
V_A_ (L)	3.8 (3.4, 6.0)	4.2 (3.5, 6.1)	3.9 (3.4, 5.6)	0.001	3.9 (3.3, 5.9)	3.9 (3.5, 5.5)	4.0 (3.6, 6.0)	0.420
V_cap_ (mL)	76 (69, 82)	91 (77, 91)	76 (67, 91)	< 0.001^*^	75 (72, 94)	82 (76, 92)	75 (67, 83)	0.015^*^
Oxygen saturation								
SpO_2_ (%)	97.0 (95.8, 97.0)	96.0 (94.8, 97.0)	96.0 (95.5, 97.0)	0.598	96.5 (96.0, 97.6)	96.0 (96.0, 97.3)	97.0 (96.8, 98.0)	0.125

Data are displayed as median (interquartile range, IQR). *D*LCO, diffusing capacity of the lung for carbon monoxide; *D*LNO, diffusing capacity of the lung for nitric oxide; *D*MCO, alveolar-capillary membrane diffusing capacity for carbon monoxide; G’, storage modulus; G”, loss modulus; SpO_2_, oxygen saturation; V_A_, alveolar volume; V_cap_, pulmonary capillary blood volume. Pulmonary gas diffusion variables were measured in SI units (mmol min^− 1^ kPa^− 1^) and converted to traditional units (mL min^− 1^ mmHg^− 1^) by multiplying with 2.987. Differences in outcome variables between the three different time points during each experimental condition (experiment A and B) were analyzed using the non-parametric Friedman test followed by a Wilcoxon-signed rank test. The Wilcoxon signed-rank test was only performed, if changes over time were significant (*P* < 0.05)

*indicates significant differences between pre- and post-exercise values based on Wilcoxon signed-rank test (*P* < 0.05)

♯ indicates significant differences between pre- and 45′ post-exercise values based on Wilcoxon signed-rank test (*P* < 0.05)

No differences in sputum rheological properties (viscoelastic moduli G’ and G”, yield stress, solids content) were found between experiments (Table 3), except differences in sputum spinnability comparing pre- versus 45′ post-exercise values between experiments. During recovery (absolute change, pre- vs. 45 min post-exercise), we noticed differences in patient reported ease of sputum expectoration, favoring experimental condition A (*P* = 0.016).

**Table 3:** Comparison of absolute changes in sputum properties, pulmonary diffusing capacity and oxygen saturation between experiments A and B (*N* = 15)

	Experiment A		Experiment B		*P*-values	
Variables	Absolute change pre- versus post-exercise	Absolute change pre- versus 45 min post-exercise	Absolute change pre- versus post-exercise	Absolute change pre- versus 45 min post exercise	Pre- versus post- exercise between A & B	Pre- versus 45 min post- exercise between A & B
Sputum properties						
G’ 1 rad s^− 1^ (Pa)	1.98 (0.1, 9.9)	− 0.27 (− 3.3, 6.8)	0.87 (− 2.8, 4.8)	3.21 (0.1, 7.2)	0.237	0.290
G’ 10 rad s^− 1^ (Pa)	2.54 (1.1, 14.2)	11.35 (−1.0, 16.5)	1.22 (− 3.9, 8.2)	2.90 (− 0.4, 15.1)	0.290	0.725
G” 1 rad s^− 1^ (Pa)	0.82 (− 1.2, 3.1)	2.40 (− 0.1, 4.1)	0.46 (− 0.2, 1.63)	1.64 (− 0.2, 3.3)	0.595	0.567
G” 10 rad s^− 1^ (Pa)	0.85 (− 0.3, 2.7)	2.52 (− 0.1, 4.0)	0.61 (− 0.6, 1.6)	1.82 (− 0.2, 3.6)	0.468	0.576
Dynamic yield stress (Pa)	0.07 (0.0, 0.3)	0.20 (0.0, 0.3)	0.01 (−0.1, 0.1)	0.10 (0.0, 0.2)	0.217	0.713
Sputum solids content (%)	0.25 (−0.1, 3.5)	0.86 (0.4, 1.9)	1.11 (−1.2, 3.4)	0.94 (− 1.6, 3.6)	0.967	0.653
Spinnability (mm)	1.00 (−0.2, 11.0)	−1.40 (− 14.3, 0.4)	−1.25 (− 3.9, 0.7)	1.20 (− 1.1, 5.7)	0.077	0.009
Ease of sputum expectoration (cm)	0.50 (− 0.2, 1.7)	0.80 (− 0.1, 1.2)	−0.20 (− 1.6, 1.3)	− 1.1 (− 1.8, 0.1)	0.276	0.016
Pulmonary diffusing capacity						
*D*LNO (mL min^− 1^ mmHg^− 1^)	4.23 (− 0.9, 8.8)	−0.90 (− 2.8, 1.4)	1.40 (− 5.7, 5.1)	−2.86 (− 7.2, 5.0)	0.152	1.000
*D*LNO/V_A_ (mL min^− 1^ mmHg^− 1^ L^− 1^)	−0.17 (− 0.6, 0.2)	− 1.04 (− 1.5, − 0.6)	−0.18 (− 0.9, 0.3)	−1.16 (− 1.8, − 0.1)	0.934	0.967
*D*LCO (mL min^− 1^.mmHg^− 1^)	2.16 (0.8, 4.5)	−0.12 (− 0.8, 0.6)	1.87 (0.1, 2.3)	−0.76 (− 2.1, 0.9)	0.110	0.351
*D*LCO/V_A_ (mL min^− 1^ mmHg^− 1^ L^− 1^)	0.28 (0.1, 0.5)	−0.09 (− 0.2, 0.1)	0.21 (0.0, 0.4)	−0.22 (− 0.3, − 0.1)	0.468	0.271
*D*LNO/*D*LCO ratio	− 0.16 (− 0.3, − 0.1)	−0.01 (− 0.2, 0.1)	−0.17 (− 0.4, 0.1)	−0.13 (− 0.2, − 0.0)	0.771	0.533
*D*MCO (mL min^− 1^ mmHg^− 1^)	1.0 (− 3.0, 3.0)	1.0 (− 5.0, 2.0)	0.0 (− 9.0, 3.0)	2.0 (− 7.0, 5.0)	0.632	0.519
V_A_ (L)	0.26 (0.0, 0.4)	0.04 (− 0.0, 0.2)	0.06 (− 0.1, 0.3)	0.03 (− 0.2, 0.2)	0.032	0.693
V_cap_ (mL)	12.0 (9.0, 17.0)	0.0 (−3.0, 3.0)	12.0 (1.0, 15.0)	−5.0 (− 11.0, 4.0)	0.135	0.114
Oxygen saturation						
SpO_2_ (%)	0.0 (− 1.5, 1.0)	0.0 (−0.8, 0.5)	0.0 (− 1.0, 0.0)	0.3 (− 0.1, 1.0)	0.983	0.378

Data are displayed as median (interquartile range, IQR). *D*LCO, diffusing capacity of the lung for carbon monoxide; *D*LNO, diffusing capacity of the lung for nitric oxide; *D*MCO, alveolar-capillary membrane diffusing capacity for carbon monoxide; G’, storage modulus; G”, loss modulus; SpO_2_, oxygen saturation; V_A_, alveolar volume; V_cap_, pulmonary capillary blood volume. Pulmonary gas diffusion variables were measured in SI units (mmol min^− 1^ kPa^− 1^) and converted to traditional units (mL min^− 1^ mmHg^− 1^) by multiplying with 2.987. Differences in outcome variables at different time points (pre-exercise versus post-exercise and pre-exercise versus 45′ post-exercise) between the two experimental conditions were analyzed using the non-parametric Mann-Whitney-U test

### Changes in pulmonary diffusing capacity

Individual raw data for *D*LNO, *D*LCO, V_A_ and V_cap_ at different time points during experiments A and B are shown in Additional file 7: Figure S3 and Additional file 8: Figure S4 in the online supplements. Time course changes in pulmonary diffusing capacity were observed during both experiments, while changes in *D*LNO and V_A_ were only observed in experimental condition A (Table 2). No differences in pulmonary diffusing capacity were found between the two experimental conditions (Table 3), except a higher V_A_ comparing post-exercise and pre-exercise changes favoring experiment A (*P* = 0.032).

## Discussion

This randomized controlled crossover study investigated acute effects of moderate intensity continuous cycling exercise versus interval cycling exercise incorporating the Flutter® device on sputum viscoelasticity (primary endpoint) and pulmonary diffusing capacity in adults with CF. This study provides three important findings. First, the addition of Flutter® to moderately intense stationary cycling has no measurable effect on sputum viscoelastic properties compared to stationary cycling alone. Second, our results highlight experimental challenges to simulate ‘coughing’ (i.e., 100 rad s^− 1^ measurements or generally high frequency measurements) in a shear rheological setup due to instrument inertia effects. Third, the increase in pulmonary diffusing capacity over time reflects an acute exercise-induced effect, not sustained post-exercise.

### Sputum rheological properties and exercise

In the present study, we found no differences in sputum viscoelasticity between the two experimental conditions, thus rejecting our initial hypothesis. Two previous studies reported altered sputum viscoelasticity after an acute bout of treadmill exercise or Flutter® and treadmill exercise compared to resting breathing in adults with CF [6, 11]. Treadmill but not cycling exercise reduced sputum viscoelasticity and trunk oscillations during treadmill running/walking have been postulated as a possible underlying mechanism [11]. One could argue that the chosen exercise mode in our study was not sufficient to improve viscoelastic properties of sputum in our patients and/or that the magnitude of effect from Flutter® was not large enough to produce greater changes in sputum viscoelastic properties compared to cycling alone. During exercise, our patients achieved approximately 47–49% of their maximal minute ventilation (90–97% of their maximal tidal volumes and 50–55% of their maximal respiratory frequencies, see Additional file 2: Table S2) suggestive of a high ventilatory demand. Higher exercise intensities (e.g., 90% of maximal heart rate) may induce significant airway narrowing [12] unlikely to be maintained by many patients when exercising for longer periods, in particular, in patients with advanced lung disease. With respect to Flutter®, our patients performed 6–10 breathing maneuvers during each of four cycles. Higher volumes of Flutter® therapy (6 cycles à 15 breaths) combined with huffing and coughing (forced expiration technique, FET) improved sputum viscoelasticity compared to resting breathing in adults with CF [6]. It is important to note that we deliberately did not follow existing CF physiotherapy recommendations [27] suggesting to individually determine the angle of the Flutter® along with the performance of forced expiratory maneuvers. In this study, we were mainly interested in mechanistic effects of airway oscillations on sputum viscoelasticity [7] on top of high ventilation during exercise. We did not implement forced expiratory maneuvers due to potential interference with our cycling protocol and pulmonary gas exchange measurements. The authors hypothesize that a more intense Flutter® therapy, together with individual adaptation of the Flutter® angle, but not the lack of forced expiratory maneuvers, could explain the absence of additional effect in our study. Given the fact that positive expiratory pressure and oscillations are thought to mechanically impact on biophysical properties of mucus (i.e., reduction of viscoelasticity), huffing and coughing following Flutter® maneuvers assist in mobilization and transport of secretions from peripheral to central airways, but should not substantially change viscoelastic properties of sputum in addition. This hypothesis is supported by an in vitro experiment demonstrating that Flutter® oscillations alone augment sputum elasticity after 15 and 30 min, respectively [7].

It is important to mention that in comparison to our experimental study design, the study by Dwyer et al. [6] compared Flutter® therapy with resting breathing (no intervention), which likely increases the chance to observe effects between experimental conditions. Nevertheless, given the high variability of sputum properties [33] and differing intra- and interindividual responses to airway clearance therapy, a “no intervention” visit would have probably provided further insights into within-patient treatment responses.

Interestingly, and in line with findings by Dwyer et al. [11], ease of sputum expectoration was higher during recovery from continuous cycling versus cycling exercise with Flutter®. This suggests that patients perceive the benefit of airway clearance therapy not immediately but during recovery from exercise. However, the clinical meaningfulness of these findings cannot be interpreted due to the lack of a minimal important difference for the visual analogue scale.

### Rheological measurements and inertia

We observed occurrence of inertia at high frequencies, causing experimental artifacts (see Additional file 9 for further discussion). King and Macklem suggested first that sputum rheology at high frequency simulates cough studying dog tracheal mucus [34]. The concept was then further elaborated suggesting that low frequency (1 rad s^− 1^) deformations are relevant when simulating ciliary transport [35] whereas high frequency (100 rad s^− 1^) deformations are more characteristic for cough clearance [36]. The 1 rad s^− 1^ / 100 rad s^− 1^ concept was initially elaborated using active microrheology but was later applied in shear rheology testing [6, 11, 37, 38]. We found a strong increase in moduli (G’, G”) and phase angles at frequencies > 10 rad s^− 1^ (Fig. 2c and d, Additional file 10: Figure S5a) and observed that sample torques deviated from the electrical torques (Additional file 10: Figure S5b), both strong indicators for inertia. The deviation of sample torque from electrical torque due to inertia is schematically represented in torque vector drawings in Additional file 10: Figure S5c. Also, we calculated the theoretical inertia limit of the rheometer, which agreed well with the observed onset of inertia. Further proof is obtained comparing our results to other findings that show a continuous trend of the dynamic moduli up to 50 Hz (≈ 314 rad s ^− 1^) using passive microrheology [32], which is not affected by inertia at frequencies below 1 MHz [39]. Data generated in previous studies using shear rheology at 100 rad s^− 1^ also showed signs of inertia [6, 11, 37, 38], revealed by a substantially increased loss modulus G” or complex modulus (G*) and high tan δ values compared to those measured at 1 rad s^− 1^. Future rheology measurements with sputum should be carefully checked for inertia, as in the worst-case scenario, false positive or false negative outcomes occur. We propose restricting frequencies to 10 rad s^− 1^ to simulate the high frequency behavior of sputum in shear rheology.

### Pulmonary diffusing capacity and exercise

In the present study, *D*LNO and *D*LCO increased during continuous cycling exercise, whereas interval cycling exercise interspersed with use of the Flutter® increased only *D*LCO but not *D*LNO, most likely due to the absence of increase in V_A_. It is well known that *D*LNO, *D*LCO, and V_cap_ increase linearly during exercise with respect to cardiac output [40]. In our study, continuous cycling increased post-exercise *D*LNO by about 5%, but the effect was not sustained during recovery. We extend previous findings reporting a remarkable increase in *D*LNO (39 ± 8% from rest using rebreathing measurements) during moderate intensity cycling exercise in CF adults, with the authors questioning the duration of beneficial effects [12]. Our data demonstrate that increase in *D*LNO is (only) an acute, exercise-induced effect, most likely the result of increased V_A_ and subsequently greater surface area for diffusion rather than improved mucociliary clearance mechanisms such as improved sputum hydration and/or viscoelastic properties.

This study has limitations. First, we did not measure expiratory flow during exercise [6, 11] to evaluate whether our experiments were sufficient to create an expiratory airflow bias, a mechanism potentially improving mucociliary clearance. Furthermore, the sputum was sheared at 50 Pa in simple shear experiments prior to spinnability measurements. The shear homogenized the samples, theoretically disrupting sputum microstructure, which could have caused the comparably low spinnability values. Finally, our study patient cohort was small, limiting the generalizability of our findings to the overall CF population.

## Conclusions

We conclude that the addition of Flutter® to moderate intensity interval cycling exercise has no measurable effect on the viscoelastic properties of sputum compared to moderate intensity continuous cycling alone. The higher pulmonary diffusing capacity represents an acute exercise-induced effect not sustained post-exercise.

## Additional files

Additional file 1: **Table S1**. Pre-exercise sputum properties, pulmonary function data and patient-reported health status at each study visit (*N* = 15). (DOCX 27 kb)

Additional file 2: **Table S2.** Comparison of cardiorespiratory variables at rest and exercise between the two experimental conditions (N = 15). (DOCX 22 kb)

Additional file 3: **Table S4.** Derived viscoelastic parameters from cystic fibrosis sputum at rest. (DOCX 23 kb)

Additional file 4: **Figure S1.** Comparison of individual raw data for G’ (storage modulus) at 1 and 10 rad.s^− 1^ at different time points during experiment A and experiment B (N = 15). (DOCX 432 kb)

Additional file 5: **Figure S2.** Comparison of individual raw data for G’ (storage modulus) at 1 and 10 rad.s^− 1^ at different time points during experiment A and experiment B (N = 14, excluding the outlier). (DOCX 453 kb)

Additional file 6: **Table S3**. Changes in sputum rheological properties during experiments A and B without one extreme outlier (*N* = 14). (DOCX 25 kb)

Additional file 7: **Figure S3.** Comparison of individual raw data for *D*LNO and *D*LCO at different time points during experiment A and experiment B (N = 15). (DOCX 468 kb)

Additional file 8: **Figure S4.** Comparison of individual raw data for alveolar volume (V_A_) and V_cap_ at different time points during experiment A and experiment B (N = 15). (DOCX 1406 kb)

Additional file 9: Instrument inertia causes artifacts in shear rheology measurements at high frequencies. (DOCX 29 kb)

Additional file 10: **Figure S5**. Instrument inertia in cystic fibrosis sputum shear rheology measurements (DOCX 978 kb)

## Abbreviations

ACT: airway clearance techniques
BMI: body mass index
CF: cystic fibrosis
CPET: cardiopulmonary exercise testing
*D*LCO: pulmonary diffusing capacity for carbon monoxide
*D*LNO: pulmonary diffusing capacity for nitric oxide
*D*LNO/*D*LCO ratio: ratio of pulmonary diffusing capacity for nitric oxide to pulmonary diffusing capacity for carbon monoxide
*D*MCO: alveolar-capillary membrane diffusing capacity for carbon monoxide
FEV_1_: forced expiratory volume in 1 s
f_R_: respiratory frequency
G*: complex modulus
G’: storage modulus
G”: loss modulus
HR: heart rate
LVE: linear viscoelasticity
PEP: positive expiratory pressure
RER: respiratory exchange ratio
SpO_2_: oxygen saturation
tan δ: loss tangent
V’E: minute ventilation
V’O_2peak_: peak oxygen consumption
V_A_: alveolar volume
V_cap_: pulmonary capillary blood volume
VT: tidal volume
η^*^: complex viscosity
