# Supplementary material for: Acute effects of combined exercise and oscillatory positive expiratory pressure therapy on sputum properties and lung diffusing capacity in cystic fibrosis: a randomized, controlled, crossover trial
Source: BMC Pulm Med. 2018 Jun 14;18:99. doi: 10.1186/s12890-018-0661-1 (PMC6000950; doi:10.1186/s12890-018-0661-1)
Supplement: Supplementary file 1 — Table S1. Pre-exercise sputum properties, pulmonary function data and patient-reported health status at each study visit (N = 15). (DOCX 27 kb) [file 12890_2018_661_MOESM1_ESM.docx]

Table S1. Pre-exercise sputum properties, pulmonary function data and patient-reported health status at each study visit (N=15)

| **Variables** | **Study visit 1** | **Study visit 2** | **Study visit 3** | ***P*-value** |
| --- | --- | --- | --- | --- |
| Time between study visits  (days after prior visit) | - | 4 (3, 4) | 3 (3, 5) | - |
| ***Sputum properties*** |  |  |  |  |
| G^’^ 1 rad s^-1^ (Pa) | 7.03 (4.58, 11.15) | 7.44 (5.53, 14.56) | 4.36 (3.45, 9.68) | 0.247 |
| G^’^ 10 rad s^-1^ (Pa) | 9.15 (6.29, 15.98) | 11.43 (7.55, 20.09) | 6.44 (5.02, 14.40) | 0.282 |
| G^’’^ 1 rad s^-1^ (Pa) | 2.13 (1.74, 3.22) | 2.52 (1.94, 5.04) | 1.80 (1.25, 3.19) | 0.549 |
| G^’’^ 10 rad s^-1^ (Pa) | 2.69 (2.21, 3.97) | 2.43 (1.83, 5.55) | 2.39 (1.76, 3.96) | 0.534 |
| Dynamic yield stress (Pa) | 0.2 (0.1, 0.3) | 0.2 (0.1, 0.3) | 0.2 (0.1, 0.3) | 0.673 |
| Sputum spinnability (mm) | 7.95 (5.83, 13.65) | 7.05 (5.78, 14.90) | 7.40 (6.50, 11.23) | 0.789 |
| Sputum solids content (%) | 4.32 (2.99, 6.43) | 4.54 (3.92, 6.38) | 5.32 (3.60, 7.36) | 0.085 |
| Ease of sputum expectoration (cm) | 6.8 (4.5, 7.9) | 8.0 (5.4, 8.8) | 7.3 (4.7, 8.3) | 0.410 |
| ***Spirometry*** |  |  |  |  |
| FEV_1_ (L) | 1.9 (1.4, 3.4) | 1.8 (1.5, 3.3) | 1.9 (1.4, 3.2) | 0.270 |
| FVC (L) | 2.8 (2.5, 4.9) | 2.9 (2.6, 4.8) | 2.8 (2.6, 4.6) | 0.330 |
| ***Pulmonary diffusing capacity*** |  |  |  |  |
| *D*LNO (mL min^-1^mmHg^-1^) | 83 (70, 137) | 81 (66, 142) | 86 (65, 152) | 0.819 |
| *D*LCO (mL min^-1^mmHg^-1^) | 22.3 (21.0, 30.2) | 22.4 (16.4, 31.9) | 22.2 (20.3, 35.4) | 0.420 |
| *D*LNO/ *D*LCO ratio | 3.8 (3.6, 4.1) | 3.7 (3.4, 4.2) | 3.8 (3.5, 4.1) | 0.799 |
| *D*MCO (mL min^-1^mmHg^-1^) | 56 (45, 106) | 52 (42, 106) | 59 (43, 112) | 0.309 |
| V_A_ (L) | 3.9 (3.4, 5.8) | 3.8 (3.2, 5.9) | 3.9 (3.4, 6.0) | 0.321 |
| V_C_ (mL) | 75.0 (71.3, 86.5) | 73.5 (69.8, 90.3) | 76.7 (69.8, 98.5) | 0.618 |
| ***Patient-reported health status*** |  |  |  |  |
| Feeling thermometer (0-100) | 80 (62, 90) | 80 (70, 90) | 80 (70, 90) | 0.215 |

Data are given as median (IQR). *D*LCO, diffusing capacity of the lung for carbon monoxide; *D*LNO, diffusing capacity of the lung for nitric oxide; *D*MCO, alveolar-capillary membrane diffusing capacity for carbon monoxide; FEV_1_, forced expiratory volume in one second; FVC, forced vital capacity; (G’, storage modulus; G’’, loss modulus); V_A_; alveolar volume; Vc, pulmonary capillary blood volume. The non-parametric Friedman test was used for comparisons between pre-exercise outcomes between the different study visits.
