# Supplementary material for: Acute effects of combined exercise and oscillatory positive expiratory pressure therapy on sputum properties and lung diffusing capacity in cystic fibrosis: a randomized, controlled, crossover trial
Source: BMC Pulm Med. 2018 Jun 14;18:99. doi: 10.1186/s12890-018-0661-1 (PMC6000950; doi:10.1186/s12890-018-0661-1)
Supplement: Supplementary file 2 — Table S2. Comparison of cardiorespiratory variables at rest and exercise between the two experimental conditions (N = 15). (DOCX 22 kb) [file 12890_2018_661_MOESM2_ESM.docx]

Table S2. Comparison of cardiorespiratory variables at rest and exercise between the two experimental conditions (N=15)

| **Variables** | **Experiment A** | | **Experiment B** | | ***P*-value** |
| --- | --- | --- | --- | --- | --- |
|  | *Rest* | *Exercise* | *Rest* | *Exercise* | *Exercise A vs. B* |
| Mechanical power (W) | - | 55 (50, 100) [44] | - | 55 (50, 100) [44] | 1.000 |
| HR (beats min^-1^) | 91 (76, 98) | 132 (126, 145) [80] | 92 (82, 100) | 128 (121, 142) [79] | 0.604 |
| V’O_2_ (L min^-1^) | 0.29 (0.24, 0.36) | 0.93 (0.79, 1.59) [61] | 0.28 (0.24, 0.38) | 0.98 (0.79, 1.46) [61] | 0.760 |
| V’CO_2_ (L min^-1^) | 0.30 (0.94, 1.12) | 0.95 (0.78, 1.69) [50] | 0.30 (0.24, 0.39) | 1.03 (0.78, 1.56) [50] | 0.861 |
| V’_E_ (L min^-1^) | 12.5 (8.8, 14.6) | 30.7 (26.3, 45.5) [49] | 12.4 (8.1, 14.1) | 30.4 (25.2, 43.2) [47] | 0.541 |
| VT (L) | 0.80 (0.55, 1.00) | 1.10 (0.96, 2.23) [91] | 0.74 (0.58, 1.05) | 1.17 (0.98, 2.37) [96] | 0.315 |
| f_R_ (breaths min^-1^) | 15.5 (13.1, 18.9) | 25.9 (20.9, 30.2) [55] | 14.8 (13.07, 20.22) | 25.8 (18.87, 26.73) [50] | <0.001 |
| SpO_2_ (%) | 99.9 (99.0, 99.9) | 98.6 (96.9, 99.6) [96] | 99.5 (99.0, 99.9) | 98.8 (98.0, 99.3) [105] | 0.790 |
| Borg dyspnea (0-10) | - | 1.5 (0.0, 3.0) [33] | - | 2.0 (0.0, 3.5) [41] | 0.249 |
| Borg leg fatigue (0-10) | - | 3.0 (2.8, 4.0) [47] | - | 2.0 (1.0, 4.0) [38] | 0.121 |

Data are given as median (IQR) and percentage of peak exercise values [%] obtained from cardiopulmonary exercise testing at the first study visit. f_R_, respiratory frequency; HR, heart rate; SpO_2_, oxygen saturation; V’CO_2_, carbon dioxide production; V’E, minute ventilation; V’O_2_, oxygen consumption; VT_,_ tidal volume. Comparisons of cardiorespiratory variables and Borg scores between the two experimental conditions were performed using the non-parametric Mann-Whitney-U test. There were no significant differences in resting cardiorespiratory parameters between the experimental conditions A and B.
