# Supplementary material for: Acute effects of combined exercise and oscillatory positive expiratory pressure therapy on sputum properties and lung diffusing capacity in cystic fibrosis: a randomized, controlled, crossover trial
Source: BMC Pulm Med. 2018 Jun 14;18:99. doi: 10.1186/s12890-018-0661-1 (PMC6000950; doi:10.1186/s12890-018-0661-1)
Supplement: Supplementary file 3 — Table S4. Derived viscoelastic parameters from cystic fibrosis sputum at rest. (DOCX 23 kb) [file 12890_2018_661_MOESM3_ESM.docx]

Table S4. Derived viscoelastic parameters from cystic fibrosis sputum at rest

| **Rheological parameter** | **Average value / range (n = 45)** |
| --- | --- |
| Linear viscoelastic regime | up to approx. 1% deformation |
| Dynamic yield point | 1.9% +- 1.4% [% deformation] |
| Dynamic yield stress | 0.23 +- 0.20 [Pa] |
| G0 | 9.6 +- 5.3 [Pa] |
| G’ (fitted) | G’ = ω^0.174+-0.003^ * 8.1 [Pa] |
| G’’ (fitted) | G’’ = ω^0.066+-0.006^ * 2.7 [Pa] |
| η* (fitted) | η* = ω^-0.838+-0.002^ * 9.1 [Pa s] |

G0, plateau modulus of the linear viscoelastic regime; G’, storage modulus; G’’, loss modulus; η*, complex viscosity; ω, angular frequency (rad s^-1^); fitted refers to a linear fit performed in the G’/G’’ vs ω and η* vs ω log-log diagram for angular frequencies between 0.1 – 10 rad s^-1^.
