# Supplementary material for: Acute effects of combined exercise and oscillatory positive expiratory pressure therapy on sputum properties and lung diffusing capacity in cystic fibrosis: a randomized, controlled, crossover trial
Source: BMC Pulm Med. 2018 Jun 14;18:99. doi: 10.1186/s12890-018-0661-1 (PMC6000950; doi:10.1186/s12890-018-0661-1)

Figure S1. Comparison of individual raw data for G’ (storage modulus) at 1 and 10 rad.s^-1^ at different time points during experiment A and experiment B (N=15).


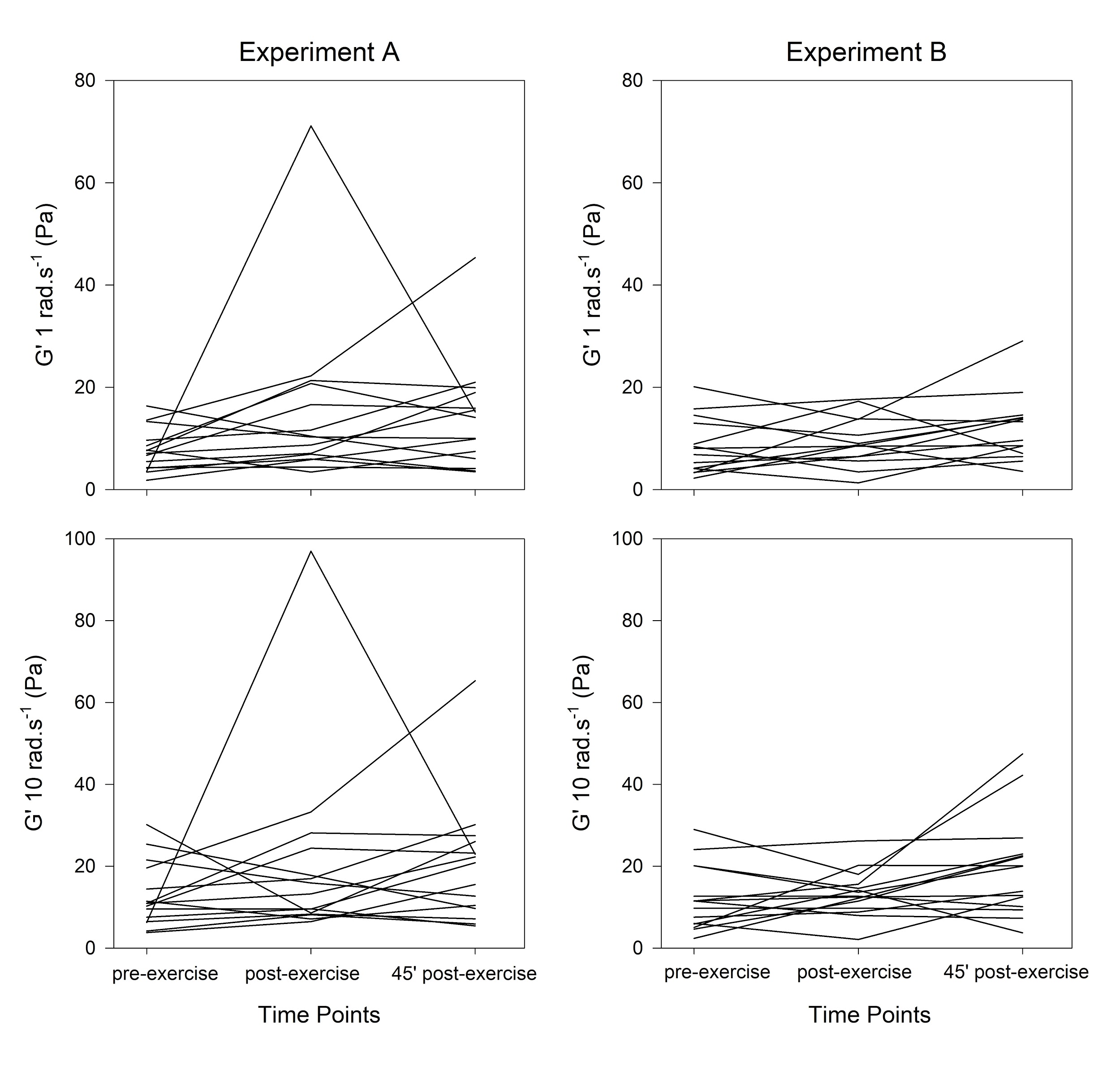

Supplement: Supplementary file 4 — Figure S1. Comparison of individual raw data for G’ (storage modulus) at 1 and 10 rad.s− 1 at different time points during experiment A and experiment B (N = 15). (DOCX 432 kb) [file 12890_2018_661_MOESM4_ESM.docx]
