# Supplementary material for: Acute effects of combined exercise and oscillatory positive expiratory pressure therapy on sputum properties and lung diffusing capacity in cystic fibrosis: a randomized, controlled, crossover trial
Source: BMC Pulm Med. 2018 Jun 14;18:99. doi: 10.1186/s12890-018-0661-1 (PMC6000950; doi:10.1186/s12890-018-0661-1)
Supplement: Supplementary file 6 — Table S3. Changes in sputum rheological properties during experiments A and B without one extreme outlier (N = 14). (DOCX 25 kb) [file 12890_2018_661_MOESM6_ESM.docx]

Table S3. Changes in sputum rheological properties during experiments A and B without one extreme outlier (N=14).

|  | **Experiment A** | | | | **Experiment B** | | | |
| --- | --- | --- | --- | --- | --- | --- | --- | --- |
| Variables | Pre-exercise | Post-exercise | 45 min post-exercise | *P*-value | Pre-exercise | Post-exercise | 45 min post-exercise | *P*-value |
| ***Sputum properties*** |  |  |  |  |  |  |  |  |
| G’ 1 rad s^-1^ (Pa) | 6.9 (4.3, 10.6) | 9.5 (6.0; 17.3) | 12.1 (5.6, 19.2) | 0.109 | 7.2 (3.9, 10.) | 8.7 (6.3, 12.6) | 11.5 (7.0, 14.3) | 0.135 |
| G’ 10 rad s^-1^ (Pa) | 10.6 (7.2, 15.7) | 14.6 (8.2, 25.3) | 18.2 (9.0, 26.4) | 0.062 | 10.6 (5.7, 14.5) | 13.1 (8.6, 16.8) | 16.9 (9.9, 22.6) | 0.223 |
| G’’ 1 rad s^-1^ (Pa) | 2.4 (1.7, 3.4) | 2.9 (2.0, 5.7) | 3.7 (1.9, 6.3) | 0.145 | 2.5 (1.2, 3.2) | 3.1 (2.2, 4.7) | 4.0 (2.4, 4.8) | 0.223 |
| G’’ 10 rad s^-1^ (Pa) | 3.0 (2.2, 4.2) | 3.5 (2.5, 6.2) | 4.1 (2.5, 6.9) | 0.145 | 3.3 (1.8, 3.9) | 3.7 (2.7, 5.3) | 4.7 (2.9, 5.7) | 0.395 |
| Dynamic yield stress (Pa) | 0.20 (0.10, 0.30) | 0.30 (0.20, 0.50) | 0.30 (0.18, 0.43) | 0.132 | 0.20 (0.10, 0.40) | 0.20 (0.15, 0.35) | 0.30 (0.20, 0.50) | 0.071 |
| Sputum solids content (%) | 5.8 (3.8, 7.7) | 6.1 (4.1, 9.1) | 6.2 (4.3, 8.3) | 0.062 | 4.3 (3.6, 5.7) | 5.9 (4.1, 7.6) | 6.2 (3.7, 8.3) | 0.395 |
| Spinnability (mm) | 6.6 (6.3, 9.9) | 7.6 (6.4, 18.5) | 7.0 (6.4, 8.3) | 0.223 | 8.5 (6.3, 14.4) | 6.2 (5.8, 8.4) | 8.8 (6.9, 20.5) | 0.161 |
| Ease of sputum expectoration (cm) | 8.2 (5.7, 8.3) | 7.5 (3.3, 9.3) | 8.2 (5.7, 9.0) | 0.173 | 7.5 (5.4, 8.8) | 6.5 (4.2, 9.2) | 5.7 (4.2, 9.3) | 0.355 |

Data are given as median (interquartile range, IQR). G’, storage modulus; G’’, loss modulus. Differences in outcome variables between the three different time points during each experimental condition (experiment A and B) were analyzed using the non-parametric Friedman test.
