# Supplementary material for: Acute effects of combined exercise and oscillatory positive expiratory pressure therapy on sputum properties and lung diffusing capacity in cystic fibrosis: a randomized, controlled, crossover trial
Source: BMC Pulm Med. 2018 Jun 14;18:99. doi: 10.1186/s12890-018-0661-1 (PMC6000950; doi:10.1186/s12890-018-0661-1)
Supplement: Supplementary file 7 — Figure S3. Comparison of individual raw data for DLNO and DLCO at different time points during experiment A and experiment B (N = 15). (DOCX 468 kb) [file 12890_2018_661_MOESM7_ESM.docx]

**
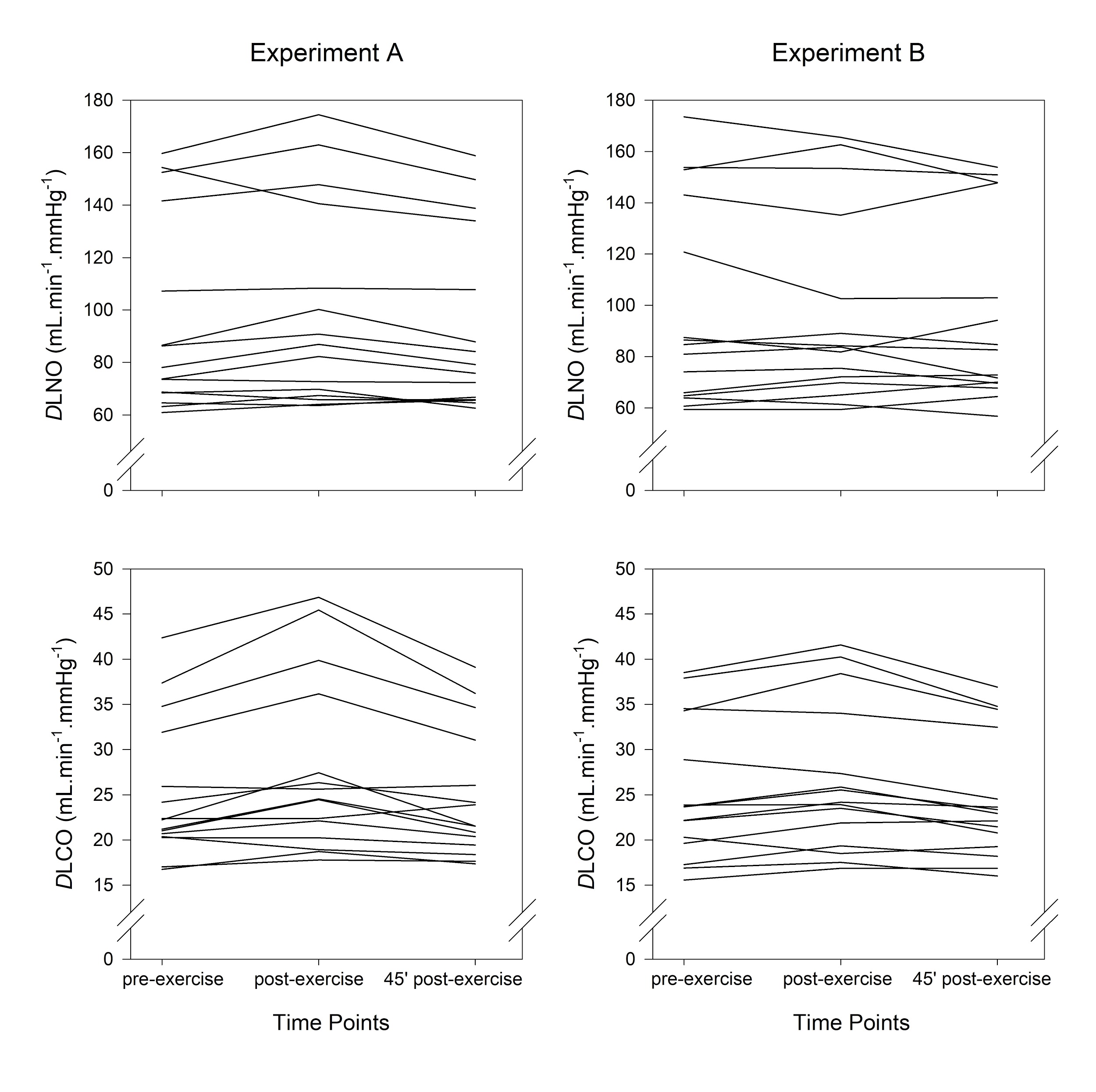
**Figure S3. Comparison of individual raw data for *D*LNO and *D*LCO at different time points during experiment A and experiment B (N=15).
