# Supplementary material for: Acute effects of combined exercise and oscillatory positive expiratory pressure therapy on sputum properties and lung diffusing capacity in cystic fibrosis: a randomized, controlled, crossover trial
Source: BMC Pulm Med. 2018 Jun 14;18:99. doi: 10.1186/s12890-018-0661-1 (PMC6000950; doi:10.1186/s12890-018-0661-1)

Figure S4. Comparison of individual raw data for alveolar volume (V_A_) and V_cap_ at different time points during experiment A and experiment B (N=15).


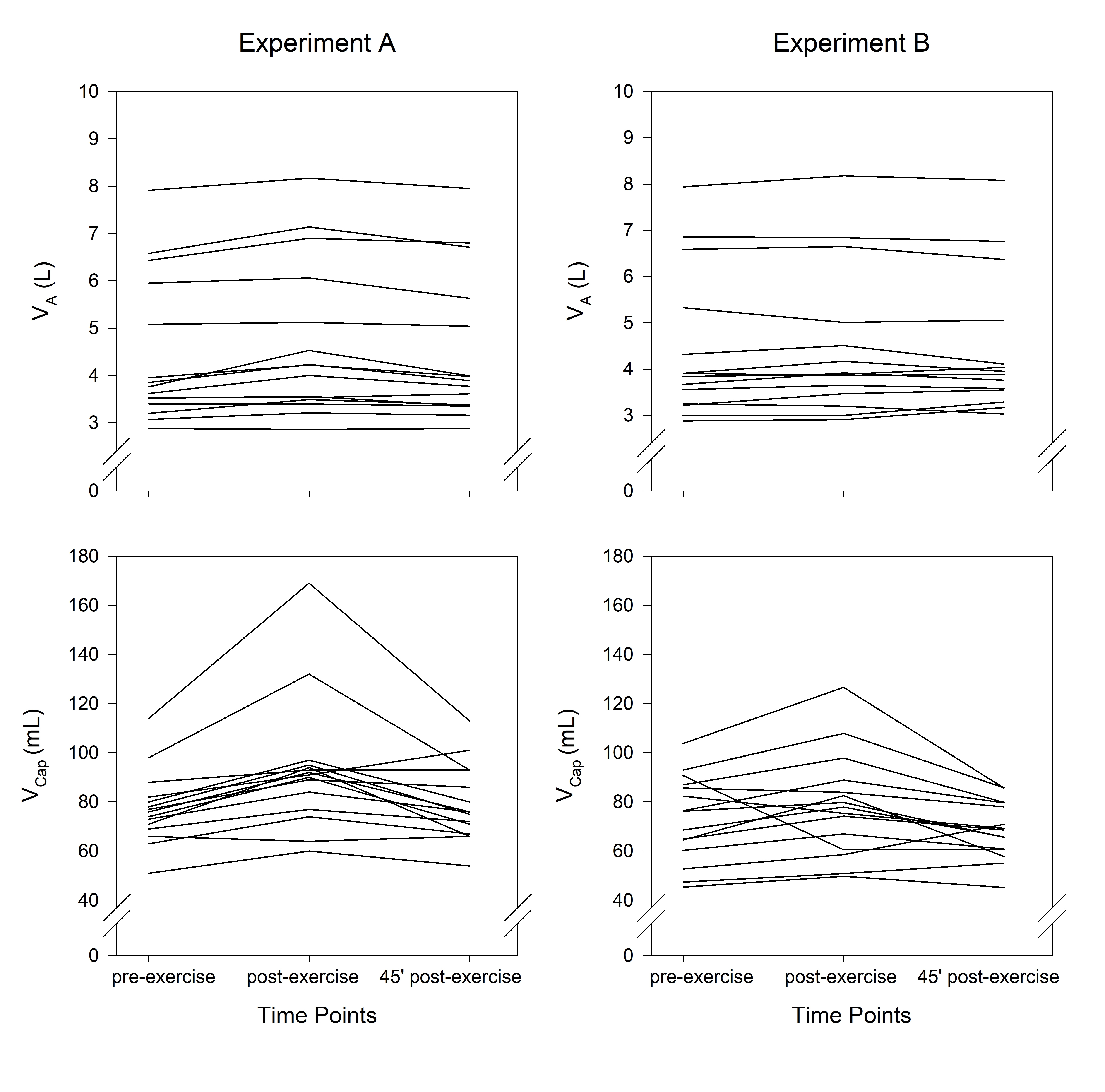

Supplement: Supplementary file 8 — Figure S4. Comparison of individual raw data for alveolar volume (VA) and Vcap at different time points during experiment A and experiment B (N = 15). (DOCX 1406 kb) [file 12890_2018_661_MOESM8_ESM.docx]
