# Supplementary material for: Acute effects of combined exercise and oscillatory positive expiratory pressure therapy on sputum properties and lung diffusing capacity in cystic fibrosis: a randomized, controlled, crossover trial
Source: BMC Pulm Med. 2018 Jun 14;18:99. doi: 10.1186/s12890-018-0661-1 (PMC6000950; doi:10.1186/s12890-018-0661-1)
Supplement: Supplementary file 9 — Instrument inertia causes artifacts in shear rheology measurements at high frequencies. (DOCX 29 kb) [file 12890_2018_661_MOESM9_ESM.docx]

**Instrument inertia causes artifacts in shear rheology measurements at high frequencies**

In shear rheology a load (torque) is applied by the rheometer and the deformation is measured to obtain material properties. In transient rheological tests, i.e., oscillatory and creep measurements some torque is also associated with acceleration and deceleration of the measuring geometry. In order to obtain good data, the “material torque” must exceed the “instrument inertia torque”, of particular importance when measuring soft biomaterials at high frequencies [1]. The effect of inertia in oscillatory shear rheology measurements with CF sputum is shown in Figure S5. The frequency sweep shows a representative baseline sample (at rest) measured over a broad frequency range (Figure S5a). At frequencies ω ≤ 10 rad s^-1^ the dynamic moduli (G’, G’’) evolve in a manner characteristic for soft biomaterials, with a storage modulus increasing slightly with frequency (G’ ∝ ω^0.17^) in a log-log plot and a loss modulus remaining almost constant (G’’ ∝ ω^0.07^). In contrast, at frequencies ω > 10 rad s^-1^ the viscous modulus and the storage modulus increase substantially (the storage modulus could also decrease substantially at this point). This putative material behavior at frequencies ω > 10 rad s^-1^ can be better interpreted when looking at the raw data generated by the rheometer (Figure S5b). The sample torque (M_sample_) and the electrical torque (M_electric_) are plotted. Note the extremely low torques in the range of hundreds of Nano-Newton Meters (10^3^ - 10^4^ nNm), still at the lower end of the dynamic range of a modern rheometer. At the point where instrument inertia starts (here around 1 rad s^-1^), the two torques (M_sample_ and M_electric_) start drifting apart [1, 2]. This phenomenon can be visualized using a schematic drawing of torque vectors at different frequencies (Figure S5c). As a rule of thumb, one can continue to measure for about one decade in frequency from the onset of inertia as inertia internally compensated by the rheometer. This can be seen in Figure S5a as the moduli are stable between 1 - 10 rad s^-1^. However, at frequencies > 10 rad s^-1^ inertia cannot be compensated anymore and affects the sample data. This is exemplified by the electrical torque increasing with a slope of 2 (log-log), a hallmark for instrument inertia [1, 2].

Inertia onset can be reduced to some extent by choosing a different measuring geometry (large over small diameter, cone-plate over plate-plate). However, when changing the geometry, other factors such as sample amount or rheological boundary conditions are frequently not satisfied. On one hand, larger measuring geometries require substantially more sample, which is often not available. On the other hand, cone-plate geometries have very small gaps (50 μm at most narrow point). However, the measuring gap should be at least 10 times larger than the smallest object in the sample in order to satisfy boundary conditions and avoid wall effects. As sputum contains cells and debris, this is possible and therefore, we suggest using a sandblasted plate-plate geometry with gaps at least 250 μm.

Our observations prompt that future high frequency rheology measurements with sputum should be carefully evaluated for inertia. Inertia creates artifacts that can look like real data, which would lead to wrong conclusions when testing effects such as exercise or drugs on patients. If authors are nevertheless interested in high frequency behavior, we propose limiting the measuring frequency to around 10 rad s^-1^. Alternatively, passive micro-rheology, which does not suffer from inertia, could be used. Also, measurements using large oscillatory deformations (large amplitude oscillatory shear - LAOS) or simple shear could be evaluated in their ability to mimic cough clearance events as coughing likely imposes large deformations on sputum.

**References**

[1] Ewoldt RH, Johnston MT, Caretta LM. Experimental Challenges of Shear Rheology: How to Avoid Bad Data. Biol Med Phys Biomed. 2015:207-41.

[2] Lauger J, Stettin H. Effects of instrument and fluid inertia in oscillatory shear in rotational rheometers. J Rheol. 2016;60:393-406.
