# Supplementary material for: Acute effects of combined exercise and oscillatory positive expiratory pressure therapy on sputum properties and lung diffusing capacity in cystic fibrosis: a randomized, controlled, crossover trial
Source: BMC Pulm Med. 2018 Jun 14;18:99. doi: 10.1186/s12890-018-0661-1 (PMC6000950; doi:10.1186/s12890-018-0661-1)
Supplement: Supplementary file 10 — Figure S5. Instrument inertia in cystic fibrosis sputum shear rheology measurements (DOCX 978 kb) [file 12890_2018_661_MOESM10_ESM.docx]

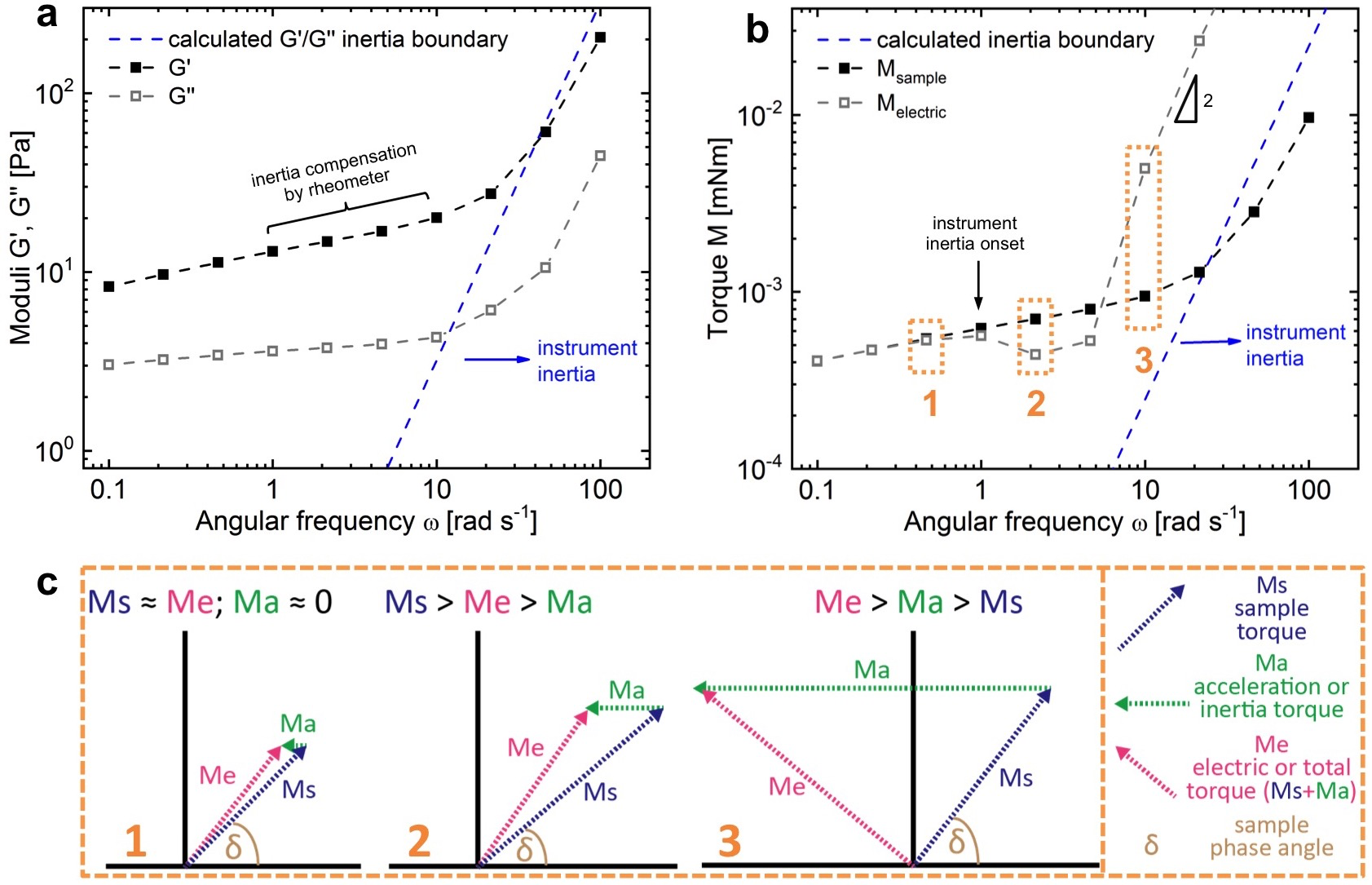


Figure S5. Instrument inertia in cystic fibrosis sputum shear rheology measurements. **(a)** Frequency sweeps a representative sputum sample depicting the storage modulus (G’) and the loss modulus (G’’). **(b)** Corresponding raw data to (a) depicting the sample torque (M_sample_) and the electrical torque (M_electric_) – corresponding to the total torque measured by the rheometer - as a function of angular frequency. Note that at frequencies ω > 10 rad s^-1^ the electrical torque starts to scale with a slope of 2 with frequency in the log-log plot, a hallmark for inertia. The blue dashed lines show the calculated inertia limit for an Anton Paar MCR (302, 502, 702) rheometer using a plate-plate 25 mm diameter (PP25) measuring geometry, which agrees well with the observed onset of instrument inertia. The calculations are based on a formula provided by Ewoldt et al. [1]. (**c**) Schematic drawing of torque vector diagrams. Torque vectors are shown for three distinct angular frequencies corresponding to boxes in (b). **1** depicts the almost complete absence of inertia, whereas **2** and **3** show different scenarios of instrument inertia affecting the total torque.

**Reference**

[1] Ewoldt RH, Johnston MT, Caretta LM. Experimental Challenges of Shear Rheology: How to Avoid Bad Data. Biol Med Phys Biomed. 2015:207-41.
